# Seed-effect modeling improves the consistency of genome-wide loss-of-function screens and identifies synthetic lethal vulnerabilities in cancer cells

**DOI:** 10.1186/s13073-017-0440-2

**Published:** 2017-06-01

**Authors:** Alok Jaiswal, Gopal Peddinti, Yevhen Akimov, Krister Wennerberg, Sergey Kuznetsov, Jing Tang, Tero Aittokallio

**Affiliations:** 1 Institute for Molecular Medicine Finland (FIMM), University of Helsinki, Helsinki, Finland; 2 Department of Mathematics and Statistics, University of Turku, Turku, Finland

**Keywords:** RNAi screening, Gene essentiality, Seed essentiality, Off-target effects, Reproducibility, Seed effects, Synthetic lethality, Cancer cells, Precision oncology

## Abstract

**Background:**

Genome-wide loss-of-function profiling is widely used for systematic identification of genetic dependencies in cancer cells; however, the poor reproducibility of RNA interference (RNAi) screens has been a major concern due to frequent off-target effects. Currently, a detailed understanding of the key factors contributing to the sub-optimal consistency is still a lacking, especially on how to improve the reliability of future RNAi screens by controlling for factors that determine their off-target propensity.

**Methods:**

We performed a systematic, quantitative analysis of the consistency between two genome-wide shRNA screens conducted on a compendium of cancer cell lines, and also compared several gene summarization methods for inferring gene essentiality from shRNA level data. We then devised novel concepts of seed essentiality and shRNA family, based on seed region sequences of shRNAs, to study in-depth the contribution of seed-mediated off-target effects to the consistency of the two screens. We further investigated two seed-sequence properties, seed pairing stability, and target abundance in terms of their capability to minimize the off-target effects in post-screening data analysis. Finally, we applied this novel methodology to identify genetic interactions and synthetic lethal partners of cancer drivers, and confirmed differential essentiality phenotypes by detailed CRISPR/Cas9 experiments.

**Results:**

Using the novel concepts of seed essentiality and shRNA family, we demonstrate how genome-wide loss-of-function profiling of a common set of cancer cell lines can be actually made fairly reproducible when considering seed-mediated off-target effects. Importantly, by excluding shRNAs having higher propensity for off-target effects, based on their seed-sequence properties, one can remove noise from the genome-wide shRNA datasets. As a translational application case, we demonstrate enhanced reproducibility of genetic interaction partners of common cancer drivers, as well as identify novel synthetic lethal partners of a major oncogenic driver, PIK3CA, supported by a complementary CRISPR/Cas9 experiment.

**Conclusions:**

We provide practical guidelines for improved design and analysis of genome-wide loss-of-function profiling and demonstrate how this novel strategy can be applied towards improved mapping of genetic dependencies of cancer cells to aid development of targeted anticancer treatments.

**Electronic supplementary material:**

The online version of this article (doi:10.1186/s13073-017-0440-2) contains supplementary material, which is available to authorized users.

## Background

RNA interference (RNAi) screening is a powerful technique for gene silencing that is widely applied for systematic profiling of loss-of-function phenotypes, for instance, in establishing gene function [1], and identifying genetic vulnerabilities in cancer cells [2–7]. Considerable efforts have been devoted to designing efficient genome-wide RNAi libraries, composed either of small interfering RNAs (siRNA) or short hairpin RNAs (shRNA), using both pooled and arrayed formats for cell-based screens [8]. While the CRISPR/Cas9 system has recently enabled genome-wide knockout screening in human cells [9–11], several technical factors, such as off-target effects [12], DNA accessibility [13], and copy number status of target genes [14, 15], may lead to increased variability of CRISPR/Cas9 phenotypic readouts. Thus, the RNAi technique remains a valuable tool for functional genomic screening, with many large-scale profiling datasets for genetic dependencies emerging in various cancer cell line panels [16–18].

However, multiple reports of high false discovery rates have reduced the promised impact of genome-wide RNAi screens [8, 18, 19], hence calling into question the reliability of the findings, usefulness of the technique, and reproducibility of the existing datasets. The relatively low hit validation rate has been notable, for instance, in the systematic identification of synthetic lethal partners for “undruggable” cancer oncogenes [20]. The concept of synthetic lethality, based on finding genetic interactions between cancer drivers and their “druggable” partners [21], was proposed as a revolutionary approach to targeted anticancer treatment [22], but so far only a few synthetic lethality-based treatments have made it to the clinic [23]. In some cases, the identified synthetic lethal hits from large-scale RNAi screens have been refuted by follow-up studies [24–26], leading to wasted drug discovery efforts and increased confusion about the reproducibility of the RNAi methodology.

The high false discovery rate observed in siRNA-based screens has often been attributed to the presence of off-target effects, mediated primarily through the “seed” region, 2–8-nucleotide positions in the guide strand of the RNAi molecule [27]. Such seed sequence-specific off-target effects result in altered expression of a large number of genes beyond the intended targets [28]. Further, down-regulated genes are enriched for seed complementary sites in the 3′ UTR region [29]. Since the seed effects are known to be inherent in genome-wide RNAi screens [30], it is likely that many of the conducted loss-of-function studies in cancer cell lines, and other cellular model systems, are also affected by the off-target effects. Although various strategies have been developed for analyzing and correcting siRNA-based screening data [31–38], what are still lacking are a comprehensive, quantitative assessment of the reproducibility of shRNA-based screens and a detailed characterization of the key factors, including seed-mediated effects, heterogeneous processing of shRNAs [39], disease models, and experimental protocols, in terms of their contribution to the sub-optimal consistency.

We present here a systematic comparison of the consistency of two genome-wide shRNA screening datasets [5–7], conducted using a pool of identical shRNA constructs from the same RNAi library across a matched panel of cancer cell lines. We demonstrate that seed-mediated off-target effects are widely prevalent in the two datasets and, in fact, significantly more consistent than the direct, intended on-target effects. In particular, we identified factors based on seed-sequence composition that significantly influenced the consistency of phenotypic outcomes in these shRNA datasets, which should be considered when designing future loss-of-function screens and their post-processing. We also apply these results in post-screening analysis to identify novel synthetic lethal partners of PIK3CA, which were consistently detected in both of the datasets, as well as confirmed by our CRISPR/Cas9 experiments, thereby demonstrating a direct clinical application towards improved mapping of functional vulnerabilities and genetic dependencies in cancer cells.

## Methods

### shRNA datasets

Achilles 2.0 and Achilles 2.4 datasets originated from a genome-wide pooled shRNA pan-cancer screen in 102 and 216 cancer cell lines, respectively [5, 6]. In both screens, each cell line was infected in quadruplicate with a lentiviral shRNA library comprising 54,020 shRNAs targeting ~11,000 genes, derived from The RNAi Consortium. The shRNA abundance was measured after allowing the cells to grow for 16 population doublings or 40 days in culture, whichever came first, and was compared to the initial DNA plasmid pool. The abundance of each shRNA constructs at both time points was measured by microarray hybridization in Achilles 2.0 and next-generation sequencing (NGS) in Achilles 2.4. Following a standard quality control (QC) and quantification pipeline, the shRNA essentiality score (shES), a measure of the effect of an shRNA on cell proliferation, was estimated using normalized fold change between initial and final time points averaged over the replicates.

The COLT-Cancer dataset consisted of a total of 72 cancer cell lines comprising three cell types: breast, pancreatic, and ovarian cancer [7]. Each cell line was screened in triplicate and three time points were assessed for overall shRNA abundance during six to eight population doublings. The shESs were estimated as the ratio of change in expression intensity of the shRNAs over population doublings.

### Gene essentiality scores

#### RIGER

Normalized enrichment scores for on-target genes were calculated by RIGER (RNAi gene enrichment ranking) as implemented in the GENE-E software package (http://www.broadinstitute.org/cancer/software/GENE-E/). Briefly, normalized shES scores from both Achilles 2.4 and COLT-cancer datasets were summarized to on-target genes by using the Kolmogorov–Smirnov statistic.

#### ATARiS

Gene-level essentiality scores were calculated using the ATARiS module as implemented in the Genepattern software [40]. Normalized shES scores from both Achilles 2.4 and COLT-cancer datasets were given as input files. Since ATARiS is dependent on the number of samples across which shRNA data are provided, we used high quality cell line data (i.e., the set of cell lines meeting QC criteria and commonly screened between Achilles 2.4 and the COLT-Cancer study). Only genes for which ATARiS was able find solutions in both datasets were considered in the correlation analysis.

#### GARP

Gene-level summary scores were calculated by averaging over the top two most essential shRNAs against an intended target gene [7]. In cases of only one shRNA per target gene, the shES score was considered as the GARP score.

#### gespeR

gespeR [36] fits a linear regression model of the shRNA–gene target relationship on shES values using elastic net regularization. Briefly, we obtained the shRNA-target relationship matrix for all 46,474 shRNAs using TargetScan [41], as suggested by the authors, except for the mixing parameter (α), which we set to 0 (i.e., ridge regression) in our analysis to obtain the gespeR-based gene essentiality score (geneES), as the default 0.5 led to numerical errors. We also reasoned that the ridge regression formulation is more suitable because our objective was to estimate geneES at the genome-wide scale for comparing the consistency between the two screens, instead of selecting the essential genes most predictive of shES.

### Seed essentiality scores

All shRNAs were grouped by the identity of the nucleotide seed sequence from positions 2–8. An illustration of the concept is presented in Additional file 1: Figure S2. A total of 9115 unique seed sequences were found in the 46,474 shRNAs commonly screened in both studies. Theoretically, the number of possible unique heptamers is 16,384. For each unique seed sequence, we averaged the shESs over all shRNAs having the same seed sequence, which we termed the seed essentiality score (seedES). We observed a wide distribution of shRNAs with identical seed sequence identity, which we termed as the shRNA family size. For instance, seedES estimates for a family size of 14 indicates that 14 shRNAs have the same seed sequence and their shESs were averaged to get the seedES value. We removed those seeds with family size >14 from analysis as there were not enough data points (<50) for comparison.

### Heptamer 12–18 essentiality score

Similar to the seedES, we considered here the heptamer sequence identity from positions 12–18 of the shRNAs, as this region in the shRNA molecule does not play a major role in target recognition [30]. All the shRNAs were grouped by identity of the hepatmer 12–18 sequence and the heptamer 12–18 essentiality score (heptamer12–18ES) was calculated by averaging over the shES of all the shRNAs in that group. The correlation between heptamer12–18ESs for matching cell lines was then calculated as a reference. We repeated the same analysis for all positions of shRNAs and calculated hepatmerESs at each interval and estimated the correlation between the screens based on these scores. Finally, the correlation estimates at all other intervals except for the seed interval, 2–8, were averaged and plotted (Additional file 1: Figure S4).

### Seed pairing stability and target abundance thresholds

We obtained seed pairing stability (SPS) and target abundance (TA) values for 7-mer heptamers from TargetScan [41], and extracted the information for the 9115 seeds that we found within the overlapping set of 46,474 shRNAs between the two studies. Strong and weak SPS thresholds as well as low and high TA thresholds were defined by the top and bottom tenth percentile of the observed distribution of SPS and TA values, respectively. In these analyses, strong SPS was defined as SPS < −9.82, weak SPS as SPS > −5.16. Low TA was defined as TA >3.72 and high TA as TA <2.89.

### Overlap of genetic interaction and synthetic lethal partners

To clean the genome-wide shRNA datasets, we removed shRNAs with strong SPS and low TA seed sequences from both the Achilles 2.4 and COLT-cancer datasets. geneESs were calculated based on GARP, both before and after cleaning. The lists of genetic interaction (GI) hits and synthetic lethal (SL) hits were defined for each driver gene in both the Achilles 2.4 and COLT-cancer datasets, separately. In these analyses, we considered the full compendium of the cell lines, 216 in Achilles 2.4 and 47 in COLT-cancer, for the detection of robust GI and SL partners, without restricting to the matching high data quality cell lines only.

### Statistical analysis

Because of the different scoring method for the shES in the two screens, rank-based Spearman correlation was used to assess the concordance of their phenotypic outcomes. A Shapiro–Wilk test was used to assess the normality of correlation distributions between the two screens. In case of normality, a paired *t*-test was used to compare the consistency calculated using different measures of essentiality: shES, geneES, seedES, or heptamer12–18ES or permuted seedES. Permuted seedES-based correlations were calculated by permuting the shRNAs and their seed mapping for 1000 times (Additional file 1: Figure S2). A non-parametric Wilcoxon rank sum test was used to compare the non-normal distributions of genes between mutated and wild-type cell lines. A Wilcoxon signed rank test was used to compare the increase in overlap of GI and SL hits before and after cleaning.

### Finding genetic interactions and synthetic lethal partners

We summarized the shES-level data to geneES-level using GARP and compared the distribution of geneESs between the mutated and wild-type cancer cell lines for each driver gene separately. The set of driver genes was taken from a recent pan-cancer study of mutational landscape in The Cancer Genome Atlas dataset [42]. We considered only those driver genes mutated in at least two cell lines in either of the datasets. The mutation status of the driver genes was obtained from CCLE [43]. In each of the datasets, we performed a two-sided Wilcoxon test to compare the differences in geneES distribution between the mutated group of cell lines and wild-type group of cell lines, and a significance threshold of 0.05 was considered for detecting GIs. For detecting SL interactions, only partners that were more essential (more negative geneES) in the mutated group of cell lines were considered using a one-sided Wilcoxon test, with a significance threshold of 0.03.

### Selection of novel SL partners of PIK3CA for experimental validation

We first selected all the novel SL partners for PIK3CA that were detected only after cleaning in both of the shRNA datasets, but not when using the original datasets. Based on improved statistical significance of GARP geneES differences between the mutated and wild-type cell lines, especially in the COLT-Cancer dataset, we selected two PIK3CA partners, HMX3 and PKN3, for in house experimental validation by CRISPR/Cas9 knockout. We confirmed that the selected genes were not reported as SL partners of PIK3CA in either Pubmed or SynLethDB [44].

### CRISPR/Cas9 knockout of HMX3 and PKN3

#### Cell lines and cell culture

Cell lines MCF10A PIK3CA (H1047R/+) and MCF10A PIK3CA (E545K/+) and a corresponding isogenic control were purchased from Horizon Discovery Group. The cells were maintained in Dulbecco’s modified Eagle medium: Nutrient Mixture F-12 (DMEM/F-12, Thermo Fisher Scientific Inc., #11330-032), supplemented with 5% horse serum (Thermo Fisher Scientific Inc., #16050-122), 20 ng/ml EGF, 0.5 mg/ml hydrocortisone, 10 μg/ml insulin, 100 ng/ml cholera toxin, 100 U/ml penicillin, and 100 μg/ml streptomycin (Thermo Fisher Scientific Inc.), in a humidified incubator with 5% CO_2_ at 37 °C.

### Lentiviral plasmid generation and packaging

Oligonucleotides encoding single-guide RNAs (sgRNAs) against HMX3 and PKN3 were ordered from SigmaAldrich (see Additional file 2: Table S1 for sequences). Lentiviral vectors for sgRNA expression were produced by cloning oligonucleotides encoding sgRNAs into LentiGuide plasmid (Addgene plasmid #52963) as described [45, 46]. 293 T cells were transfected with LentiGuide or LentiCas9 (Addgene plasmid #52962) lentiviral plasmids and packaging plasmids pCMV-VSV-G (Addgene plasmid #8454) and pCMV-dR8.2 dvpr (Addgene plasmid #8455) [3] using Lipofectamine 2000 (Thermo Fisher Scientific) transfection reagent. Supernatants were collected on the second day after transfection.

### Generation of Cas9 expressing cell lines

Cells were seeded at a density of 5 × 10^4^ cells/cm^2^ in 96-well plate format; after 2 h seeding culture medium was changed to medium containing lentiviral particles (lentiCas9, MOI = 5) and polybrene (8 μg ml^−1^). The next day, the medium was replaced with medium containing blasticidine (6 μg/ml) and cells were selected for 7 days.

### Knock-out cell line generation and proliferation assay

Cas9-expressing cell lines were seeded in a 96-well plate format (1000 cells/well) and incubated with sgRNA expressing lentivirus particles (MOI = 20) and polybrene (8 μg/ml). The next day, the medium was changed for standard growth medium. Cells were allowed to grow for 5 days and growth inhibition was measured with a CellTiter-Glo Luminescent Cell Viability Assay (Promega Inc.)

## Results

### Summary of the screening datasets and rationale for their comparison

**Fig. 1:**
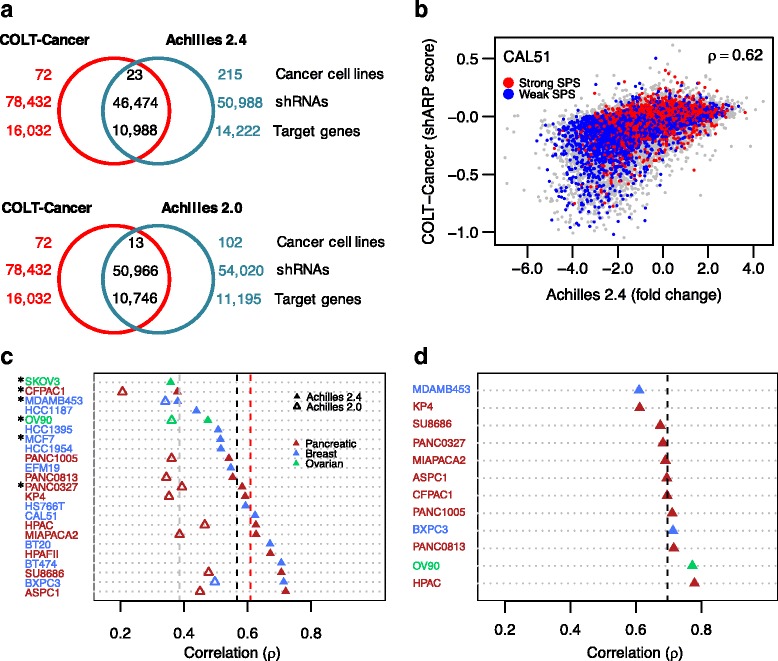
Baseline reproducibility between the Project Achilles and COLT-Cancer genome-wide shRNA screens. **a** Overlap in shRNAs, target genes, and cell lines screened in the Achilles and COLT-Cancer projects. Based on sequence identity, we found 46,474 shRNAs were commonly profiled in Achilles 2.4 and COLT-Cancer (top Venn diagram); based on The RNAi Consortium clone identifier, 50,966 shRNAs were commonly profiled in Achilles 2.0 and COLT-Cancer (bottom Venn diagram). **b** An example scatterplot of shRNA essentiality scores (shES) in Achilles 2.4 and COLT-Cancer studies across overlapping shRNAs profiled in the CAL51 cell line. The between-study consistency was assessed using Spearman rank correlation (ρ). The *red* and *blue dots* highlight those shRNAs having strong and weak seed pairing stability (SPS), respectively (see “Methods” for detailed description). **c** Inter-study correlation (ρ) for shES across matched cell lines between Achilles 2.4, Achilles 2.0, and COLT-Cancer studies. The *grey dashed line* indicates average correlation (ρ = 0.38) over the 13 cell lines between Achilles 2.0 and COLT-Cancer; the *black dashed line* average correlation (ρ = 0.57) over the 23 cell lines between Achilles 2.4 and COLT-Cancer; and the *red dashed line* average correlation (ρ = 0.61) over the 17 high data quality cell lines between Achilles 2.4 and COLT-Cancer (*asterisks* indicate cell lines with low replicate correlation ρ_rep_ < 0.5). **d** Intra-study correlation (ρ) for shES between Achilles 2.0 and 2.4. The *black dashed line* indicates average correlation over the 12 matching cell lines (ρ = 0.70). The baseline consistency between the two screens was moderate based on the shES provided in the two studies; the Achilles study scores the shRNA essentiality using normalized fold changes between initial and final time points, averaged over the replicates, whereas the COLT-cancer study uses the so-called shARP score, which is estimated as the ratio of change in expression intensity of the shRNAs over population doublings

We made use of genome-wide shRNA screens in a large panel of cancer cell lines conducted at two different laboratories, namely, Project Achilles study [5, 6] and COLT-Cancer study [7]. The Achilles datasets were generated using a genome-wide pooled shRNA screen in a pan-cancer cell line panel (Fig. 1a). Achilles 2.4 is an extension of Achilles 2.0 with screening of additional cell lines, totaling 216. Quantification of shRNA abundance at different time points was based on microarray hybridization in Achilles 2.0, and NGS in Achilles 2.4. The COLT-Cancer dataset generated from a genome-wide shRNA screen on 72 pan-cancer cell lines had an overlap of 13 and 23 cell lines with Achilles 2.0 and 2.4, respectively (Fig. 1a). In COLT-Cancer, shRNA abundance was measured by microarray hybridization in at least three time points during growth phase. Both the Achilles and COLT screens utilized the same shRNAs from The RNAi Consortium library. The raw data were deconvoluted and processed further to estimate the effect of each individual shRNA on cell proliferation (see “Methods” for details).

The two datasets provide a high-coverage and high-quality matched resource for our comparative study in terms of the use of identical shRNA libraries and similar experimental protocols (Fig. 1a). Technical differences in the screens include the estimation of shRNA abundance, the number of population doublings allowed between initial and final readouts, and quantification of shES, i.e., the quantitative estimate of the phenotypic effect of an individual shRNA in a particular cell line; the Achilles screens measured fold-change of shRNA abundance between the initial and final time points, whereas the COLT-Cancer study measured the slope of dropout of shRNAs over different time points (the so-called shARP score). Such technical differences, unless corrected for, may lead to suboptimal consistency between the studies (Fig. 1b). However, we reasoned that the substantial overlap in the shRNAs screened across the matched cell lines in the two studies provides a solid basis to perform a quantitative assessment of between-study consistency and explore ways for improving it by taking into account especially the seed effects.

### Moderate baseline reproducibility in genome-wide shRNA screens

We observed only a moderate consistency for shESs between the Achilles 2.4 and COLT-Cancer datasets, showing extensive variation across the 23 matched cell lines (average rank correlation ρ = 0.57, range = 0.36–0.72; Fig. 1c). Notably, the consistency between Achilles 2.0 and COLT-Cancer was even poorer among the 13 common cell lines, despite their use of the same shRNA abundance quantification platform (ρ = 0.37, range = 0.20–0.49, paired *t*-test *p* = 6.07 × 10^−09^). Reassuringly, the intra-study reproducibility among the 12 matched cell lines between Achilles 2.0 and 2.4 was higher (ρ = 0.70, range = 0.61–0.78; Fig. 1d). However, this is still far from ideal technical reproducibility as the only major difference between Achilles 2.0 and 2.4 was the method of quantification of shRNA abundance, microarray hybridization, or NGS. Since NGS data are known to be more reliable compared to array-based measurements [47], we focused only on Achilles 2.4 and COLT-Cancer datasets in the subsequent analyses.

To understand the factors behind the observed variability in correlation for identical cell lines, we first investigated whether data quality affected the overall consistency between the two screens. The Achilles 2.4 dataset was preprocessed and its QC already performed, requiring no further quality adjustments [6]. From the COLT-Cancer study, we excluded a subset of six cell lines with low correlation between replicates (ρ_rep_ < 0.5; marked with asterisks in Fig. 1c), which also showed significantly lower consistency between the two screens (average ρ = 0.44, Student’s *t*-test *p* = 0.005). The remaining set of 17 high data quality common cell lines resulted in slightly increased between-study consistency (average ρ = 0.61; Fig. 1c). As expected, the pairwise correlation of each cell line with the complementary set of non-matching cell lines was systematically lower than the correlation of identical cell lines between the two screens (average ρ = 0.42, Wilcoxon rank sum test, *p* < 1 × 10^−9^; Additional file 1: Figure S1), confirming that the identity of the cell line, i.e., the genetic background, plays a major role in the consistency of phenotypic effects of shRNAs.

### Decreased consistency in intended on-target geneESs

**Fig. 2:**
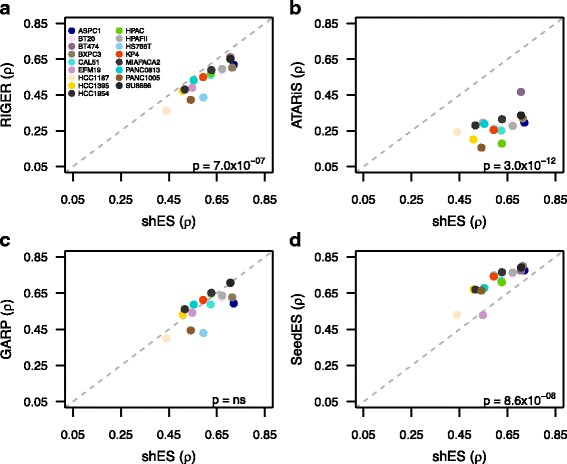
Reproducibility of the genome-wide screens at the level of shRNAs, on-target genes, and off-target seeds. Comparison of rank correlation (ρ) between the two screens over the data from 17 high-quality cell lines, where each panel compares the between-study correlation of shRNA essentiality scores (*shES*, *x-axis*) against the correlation calculated based on **a** RIGER-based gene essentiality scores (geneES), **b** ATARiS-based geneES, **c** GARP-based geneES, and **d** seed essentiality scores (*SeedES*), calculated with shRNA family size larger than 5. The on-target gene essentiality scores (**a–c**) did not improve the consistency beyond the shES-level comparison, whereas accounting for off-target effects based on SeedES improved the consistency among the matching cell lines (**d**). Statistical significance of correlation differences was assessed with paired *t*-test

To study the consistency at the level of on-target genes, we summarized the shES to gene-level estimates, the so-called gene essentiality score (geneES). More specifically, we calculated geneESs using a variety of existing gene summarization methods: RIGER [5], GARP [7], ATARiS [32] (see “Methods”). Surprisingly, the RIGER-based geneES resulted in decreased rank correlation between matched cell lines compared to that of shES (ρ = 0.54, range = 0.36–0.66, paired *t*-test *p* = 7.0 × 10^−07^; Fig. 2a). Similarly, there was an even sharper decline in the correlation with the ATARiS-based geneES (ρ = 0.28, range = 0.16–0.47, paired t-test *p* = 3.0 × 10^−12^; Fig. 2b). In contrast, we did not observe a significant decrease in the correlation based on GARP-based geneES (ρ = 0.58, range = 0.40–0.71, paired t-test *p* = 0.08; Fig. 2c). Taken together, the standard approach of summarizing the phenotypic effects of shRNA by their intended on-target gene did not lead to an increase in consistency between the two screens when compared to the shRNA level consistency.

### Increased consistency after accounting for seed-mediated off-target effects

We next investigated whether analyzing the shRNA datasets by taking into account the seed-mediated effects could lead to an increased consistency between the two screens, as was observed in a recent study [30]. To that end, shRNAs common to both the datasets were first grouped based on the heptamer nucleotide sequence identity at seed region (nucleotides 2–8) of the guide strand (Additional file 1: Figure S2). We then calculated the average shES of all the shRNAs having the same seed sequence, which we term the *seed essentiality score* (seedES). seedES is a seed-centric concept of shRNAs, analogous to microRNA (miRNA) families, in which several miRNAs having the same partial seed sequence or full sequence or structural configuration are grouped into a miRNA family [48], suggesting a similar function due to a shared profile of target genes. Similarly, we hypothesized that seedES should provide a quantitative estimate of the phenotypic effect based on a group of shRNAs having identical seed sequence, thus belonging to the same seed family. Although the specific effects of each individual shRNA in a seed family may differ in terms of the target gene profile, we reasoned that the seedES of a seed family is likely to capture the essentiality signal of the shared off-target profile, which may be more reproducible than the traditional on-target geneESs.

Similar to the design principles of genome-wide shRNA libraries, which often have five shRNAs per intended target gene, we initially restricted the analysis to seedES calculated for seed family sizes larger than five sRNAs. Interestingly, we observed significantly higher correlation between the two screens when analyzed based on the seedES (ρ = 0.71, range = 0.53–0.80, paired t-test *p* = 8.6 × 10^−08^; Fig. 2d). The correlation based on all shRNA family sizes also showed an improvement (ρ = 0.64, range = 0.41–0.74, paired *t*-test *p* = 0.007; Additional file 1: Figure S3a), but not so strong, perhaps due to a large proportion of smaller shRNA families. We further challenged these observations by repeating the same analysis for nucleotide positions 12–18 of the guide shRNA. Similar to seedES, we calculated heptamer12–18ES by averaging over shRNAs having identical nucleotide sequence at positions 12–18 (Additional file 1: Figure S2), but this did not lead to an improvement in correlation between the two screens (ρ = 0.62, range = 0.34–0.73, paired *t*-test *p* = 0.14; Additional file 1: Figure S3b). Increased correlation based on seedES indicates that the phenotypic effects in these two screens are due not only to on-target effects but, more importantly, also to the seed region-mediated off-target effects.

### Between-study consistency increases with increasing shRNA family size

**Fig. 3:**
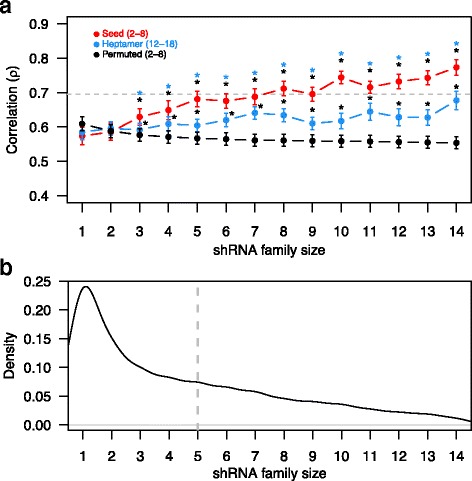
Reproducibility of the seed essentiality scores with increasing shRNA family size of seed sequences. **a** Average rank correlation (ρ), with standard error of mean over the 17 high data quality cell lines (*error bars*), calculated based on seed essentiality score (SeedES) as a function of shRNA family size (*x-axis*). shRNAs sharing the same seed sequence belong to the same shRNA family. The *red trace* indicates the observed correlation based on seed region. The *blue trace* indicates the correlation based on heptamer12–18ES for positions 12–18. The *black trace* indicates correlations based on 1000 permutations over the seed—shRNA mapping (see “Methods” for details). The *gray dotted line* indicates the intra-study correlation for shES between Achilles 2.0 and 2.4 (ρ = 0.70). SeedES-based inter-study correlation reached its maximum at family size of 14 (ρ = 0.77), suggesting that the consistency between the studies increases when off-target effects are more accurately estimated using larger family size. *Asterisks* indicate statistically significant differences in correlations (*p* < 0.05, paired *t*-test), and their colors indicate the distribution against which the comparison was done. **b** Density distribution of shRNA family size of overlapping shRNAs profiled in the two shRNA screens. Family sizes with greater than 50 unique seeds were considered in the analysis. The *gray dotted line* indicates the shRNA family of size 5

To further analyze the effect of seed family size on the between-study consistency, we divided the two datasets according to the number of shRNAs per seed family and then calculated the correlation of seedES for each seed family size among the matched cell line high-quality data. Notably, we observed that the average correlation increased with increasing family size; in particular, at shRNA family size of 14, the average correlation increased beyond the intra-study consistency observed in the Achilles study (ρ = 0.77 versus ρ = 0.70, Wilcoxon rank sum test *p* = 0.001; Fig. 3a). In contrast, when we again performed the same analysis based on the 12–18-nucleotide region of shRNA sequence, the increase in correlation was not so strong (Fig. 3a). We also noted that the correlation based on all possible positions of 7-mer length over the shRNA sequence was lower than the correlation based on the seedES (Additional file 1: Figure S4).

To further challenge the observed increase in correlation based on the seed region, we permuted the seed sequences for all shES data points in the whole dataset (see “Methods” and Additional file 1: Figure S2 for details) and checked whether the correlation based on permuted seedES was of similar strength. As expected, we did not observe an increase in correlation in the permuted datasets (Fig. 3a). These results confirm that the seed region-mediated off-target effects are consistent between identical cell lines in the two shRNA screens, and that increasingly accurate estimation of seed-mediated off-target effects can be obtained by averaging over multiple shRNAs, provided that the family size is large enough.

It has previously been observed that shRNAs are processed heterogeneously by Dicer [39]. Further, shRNAs may have various duplex RNAs as final products with a different starting position for guide strands, and therefore different seed sequences may also contribute to their off-target activity. Accordingly, we studied whether the increase in correlation with increasing shRNA family size at seed positions 2–8 is also observed if other positions of the shRNA are considered as a seed sequence. Indeed, we observed a similar trend of increase in the correlation between the two screens at other positions of the guide strand sequence (Additional file 1: Figure S5), especially in the 5′ end, suggesting shRNA processing makes a profound contribution to the observed variability between the screens.

### Effect of SPS and TA on the consistency

Because seed-mediated effects influence the consistency of the two shRNA screens so prominently, we next examined whether there are seed properties indicative of lower phenotypic consistency of shRNAs, which therefore could be used for cleaning up the current shRNA screening datasets. Previous literature suggests that thermodynamic stability of duplex formation between the seed region of siRNAs and target mRNA is a major determinant of their targeting proficiency, and hence the off-target activity of siRNAs [41, 49]. Reporter activity studies have shown that a strong pairing leads to stronger repression of bound target and hence proficient down-regulation of off-target transcripts [49]. We utilized SPS here as a measure of thermodynamic stability calculated for heptamers after taking into account biochemical parameters and base composition [41]. Another important property that also determines the targeting proficiency of shRNAs is TA, i.e., the availability of transcripts for pairing based on seed complementarity [41, 50].

**Fig. 4:**
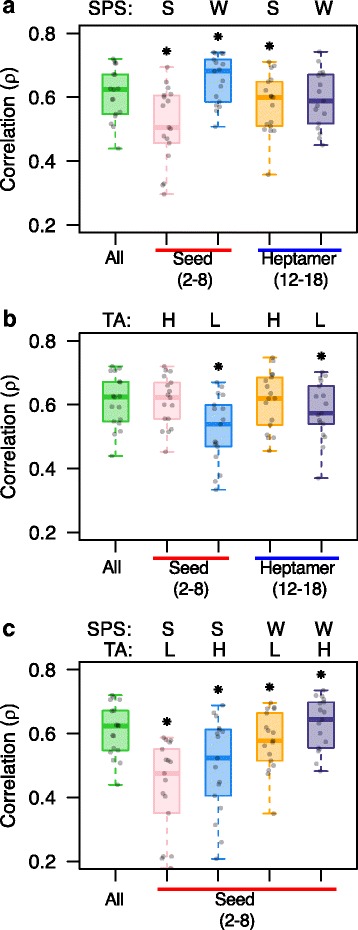
Reproducibility of the genome-wide shRNA screens after accounting for seed sequence properties. Two seed sequence properties were investigated: seed pairing stability (*SPS*) and target site abundance (*TA*). Rank correlation (ρ) over the 17 high data quality cell lines for shES of shRNAs **a** with strong (*S*) or weak (*W*) SPS, **b** with low (*L*) or high (*H*) TA, or **c** combined. Correlation for shES of shRNAs with position 12–18 heptamers after the same categorization is also shown as a reference. shRNAs with higher off-target seed sequence proficiency (i.e., strong SPS and low TA) show decreased consistency between the two studies. *Asterisks* denote statistically significant differences in correlation (*p* < 0.05, paired *t*-test). Strong SPS was defined as the top 10% percentile (SPS < −9.82), and weak SPS as the bottom 10% percentile (SPS > −5.16). Low TA >3.72 and high TA <2.89 were defined similarly, as shown at the top of each panel

Using predicted SPS and TA levels for 16,384 heptamers obtained from TargetScan [41, 51], we investigated whether these factors influenced the consistency between the two screens. Interestingly, correlation of shESs in the high data quality cell lines for the subset of shRNAs having stronger SPS seed sequences was significantly lower than that of the entire set of overlapping shRNAs (ρ = 0.51, paired *t*-test *p* = 4.8 × 10^−06^; Fig. 4a). In contrast, for shRNAs having weaker SPS seed sequences, we observed a significant increase in correlation (ρ = 0.65, paired t-test *p* = 7.0 × 10^−06^; Fig. 4a). Similarly, the correlation decreased significantly for low TA shRNAs (ρ = 0.52, paired t-test *p* = 3.3 × 10^−07^; Fig. 4b), whereas there was no shift in correlation distribution for high TA shRNAs. We again tested the validity of these observations by re-analyzing the dataset based on SPS and TA properties of heptamers from the 12–18-nucleotide region of the shRNA sequence, but did not observe a similar magnitude of change in the consistency (Fig. 4a, b). Further, we explored the inter-relationship between SPS and TA by categorizing shRNAs into stronger or weaker SPS in combination with low or high TA and found that the seed-duplex formation is more likely to influence the off-target proficiency compared to the availability of target mRNAs (Fig. 4c). These analyses suggest that when the off-target activity of a shRNA is more dominant than the on-target activity, the estimated shES is likely to be inaccurate, and therefore the consistency decreases. In contrast, when the on-target activity is more dominant, the shES provides an accurate estimate of the phenotypic effect of such shRNAs through its intended target gene.

### Improved reproducibility of GI partners of cancer drivers

An important biomedical application of genome-wide RNAi screens is to identify, often in a large compendium of cancer cell lines, what are the unique differences in genetic dependencies of cancer cells with a specific genetic background (e.g., those harboring driver mutation versus wild-type cells). Such differential gene essentialities are also known as synthetic lethal (SL) interactions, when they lie in the negative end of the genetic interaction (GI) phenotypic spectrum, and are therefore important for anticancer treatment opportunities. In contrast, positive genetic interactions are likely to contribute to the fitness advantage of cancer cells during disease progression. We therefore sought to find reproducible positive and negative GI partners of major cancer driver genes [42], which are consistently detected in the two independent shRNA screens (see “Methods” for details).

Since accurate estimation of gene essentiality is of more practical interest than seed-level relationships in the genetic interaction analyses, we investigated whether cleaning the datasets by removing shRNAs having seeds with a high propensity for off-target activity (i.e., strong SPS and low TA values) could increase the consistency at the geneES level. In these analyses we used the GARP-based geneES as it did not lead to a decrease in consistency compared to the shES-based consistency (Fig. 2c). Indeed, we observed that the geneES correlation of the shRNA screens improved significantly after cleaning the datasets (average ρ = 0.63 after cleaning versus ρ = 0.58 before cleaning, paired t-test *p* = 1.7 × 10^−08^), suggesting an improvement in the inference of gene essentiality after accounting for the seed-mediated off-target effects.

**Fig. 5:**
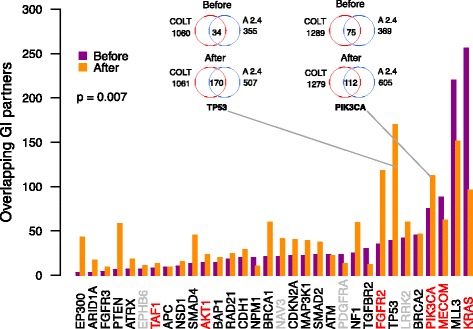
Reproducibility of genetic interaction partners of cancer drivers before and after cleaning the shRNA datasets. The number of overlapping genetic interaction (*GI*) partners of major cancer driver genes commonly detected in both shRNA datasets, before and after their cleaning by removal of shRNAs with a high tendency for off-target seed effects (defined by SPS and TA seed sequence properties; see Fig. 4 legend). The cleaning resulted in improved consistency of GI detection (*p* = 0.007, one-sided Wilcoxon signed rank test). The pan-cancer GI partners for each driver were defined based on statistical comparison of the geneES between mutated and non-mutated cancer cell lines (*p* < 0.05, Wilcoxon rank sum test). “*A 2.4*” indicates the Achilles 2.4 study. The Venn diagrams illustrate the number of overlapping GI partners of TP53 and PIK3CA, as examples of loss-of-function (LoF) and activating driver mutations, respectively. The LoF (*black*), activating (*red*), and unclassified (*grey*) status of the driver mutations was extracted from the IntoGen platform (https://www.intogen.org/)

For detecting GI partners, we performed statistical testing of the difference in GARP-based geneES phenotypes between mutated and wild-type cell lines for each driver gene in both studies separately. We did not limit these analyses to the high data quality cell lines only because we wanted to identify robust genetic interaction partners of the driver genes that are consistent across the variable cell types (so-called pan-cancer GIs). Notably, we found a statistically significant increase in the overlap of identified GI partners between the two datasets after cleaning for many well-established cancer driver genes (one-sided Wilcoxon signed rank test *p* = 0.007; Fig. 5), suggesting that cleaning the datasets by removing shRNAs with high off-target propensity can help us to identify more reliable genotype-specific dependencies of cancer cells. We also observed after cleaning a trend of increases in the overlap of SL partners for most driver genes, including KRAS (Additional file 1: Figure S6).

### CRISPR/Cas9 validation of novel synthetic lethal partners of PIK3CA

Finally, we experimentally tested whether our analytic approach for cleaning the shRNA datasets could lead to the identification of novel SL partners that would not have been detected without taking into account the seed-mediated off-target effects. We chose to study the SL partners of PIK3CA, as it is a frequently mutated oncogenic driver in many cancers; in particular, the PI3K pathway is a promising target for development of targeted therapies against breast tumors [52]. We selected two predicted SL partners of PIK3CA (Fig. 5), protein kinase PKN3 and the DNA binding transcription factor HMX3, which were consistently detected in both the Achilles 2.4 and COLT-cancer datasets with improved statistical significance after cleaning (Additional file 1: Figure S7; see “Methods” for details of the selection criteria).

**Fig. 6:**
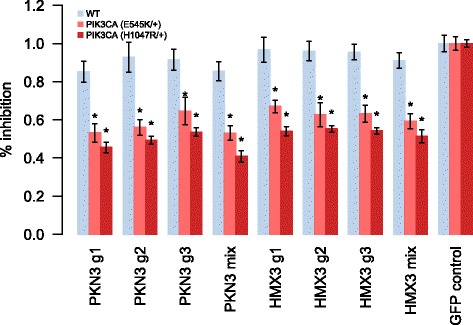
Growth inhibition of MCF10A cells with PIK3CA driver mutations and HMX3 and PKN3 knockouts. Percentage growth inhibition in MCF10A cells harboring PIK3CA (E545K/+) and PIK3CA (H1047R/+) mutation and their corresponding isogenic control (*WT*). The predicted synthetic lethal partners of PIK3CA, HMX3 and PKN3, were knocked out using three independent sgRNAs, and their mix by CRISPR/Cas9 (Additional file 2: Table S1). sgRNA against green fluorescent protein (*GFP*) was used as a negative control. The intensity data for eight replicates was averaged and scaled by the mean intensity of the GFP control in each cell line, and plotted along with the standard error of the mean (*error bars*). *Asterisks* denote statistically significant differences in the intensity values compared to wild-type MCF10A (*p* < 0.05, Student’s *t*-test)

Using MCF10A as a model system, we tested the combinatorial SL interaction strength of PIK3CA–PKN3 and PIK3CA–HMX3 pairs with CRISPR/Cas9, as we reasoned that the true SL interactions should be detectable by two complementary loss-of-function techniques (RNAi and CRISPR). Using three lentivirally delivered sgRNAs to knock out the selected genes in two isogenic MCF10A cell lines, mutated for PIK3CA either at E545K or H1074R, we observed a systematically lower rate of proliferation in the mutated cells compared to the wild-type cells (Fig. 6), hence confirming a true SL interaction with the PIK3CA oncogene. This proof-of-concept study suggests that proper modeling of the seed-mediated effects in genome-wide shRNA screens can not only lead to identification of more reproducible, pan-cancer GIs, but also enables identification of novel, context-specific SL partners of major cancer drivers.

## Discussion

This study addresses two critical challenges related to the suboptimal reproducibility of the current loss-of-function screens. First, we systematically investigated the factors behind the observed variability in genome-wide shRNA screens and provide practical means to increase their consistency in the future. In particular, based on the concepts of seed essentiality and shRNA family, we demonstrate that the consistency between shRNA screens is significantly higher for seed-mediated off-target effects compared to the intended on-target effects. As such, this suggests that reproducible seed effects are pervasive in genome-wide shRNA screens, although we also observed a moderate level of consistency for the on-target effects. Second, we provide straightforward procedures for the improved analysis of already conducted genome-wide RNAi screening efforts to extract the most reproducible biological information from the existing datasets. Towards that end, we identified shRNAs that are associated with a higher likelihood of off-target effects, based on the properties of thermodynamic stability and target abundance of their seed sequences. Such shRNAs consequently contribute to a noisy phenotype and, therefore, to inconsistent gene essentiality estimates. Removing such shRNAs with off-target propensity in the post-processing of genome-wide shRNA screens led to improved reproducibility of genetic interactions and synthetic lethal partners of major cancer driver genes.

Consistent with a previous study [30], we also found that the consistency between RNAi screens increases when analyzed based on seed essentiality. However, we observed an even higher level of correlation between the genome-wide shRNA screens in a matched panel of cancer cell lines compared to a previous study [30] that explored the consistency of genome-wide siRNA screens to find host factors required for infection of pathogens. In particular, we show the consistency based on the seed essentiality scores can increase up to 77% between the two independent shRNA screens. This is significantly higher than the within-Achilles study correlation between the shRNA-level essentiality scores (ρ = 0.70), which was considered the maximum level of consistency that can be achieved for genome-wide shRNA screens when using the same set of shRNAs. Since Achilles 2.4 and Achilles 2.0 differed only in their method for quantification of shRNA abundance, the observed within-study variation is likely due to the assay procedure and measurement noise. Importantly, we also observed an increase in correlation between the two screens based on other seed positions of the shRNA guide strand sequence, suggesting that heterogeneous processing of shRNAs is likely to contribute substantially to the variation of phenotypic outcomes in shRNA screens, which further complicates the deconvolution of off-target effects when estimating gene level activity. Further, we also confirmed that our observations are generalizable to other datasets by analyzing the consistency between Achilles 2.4 and the Breast Functional Genomics dataset [16], produced from an independent genome-wide shRNA screen in a collection of breast cancer cell lines (Additional file 1: Figures S10–S13).

In contrast to previous studies that have reported poor reproducibility of genome-wide RNAi screens [18, 19, 53], we found a moderately consistent signal already in shRNA-level data (ρ = 0.61). This improved consistency was achieved by using a common panel of cancer cell lines screened using the same RNAi library, as well as proper concordance metrics, such as genome-wide rank correlation, that consider the whole spectrum of phenotypic effects, instead of focusing on the top hits only. The current methods for summarizing shES into geneES, which do not take into account the seed-mediated off-target effects, were not able to fully extract the reproducible signal from the shRNA data, thereby leading to suboptimal consistency. We also tried the recent gespeR method [36] that models the shRNA–target gene relationships based on the seed sequence complementarity to the 3′ UTR of transcripts to estimate geneESs. After tailoring its parameters for these datasets (see “Methods”), it provided a consistency similar to using shESs (ρ = 0.66), further supporting the importance of accounting for the seed effects. Only after using the seedES modeling did we reach the maximal consistency between the two technically similar shRNA screens (ρ = 0.77). However, although the gene-level phenotypic estimates derived from gespeR [36] were correlated between the two datasets, we found that the estimates for the gold standard core-essential genes [54] were not that different from the overall phenotypic distribution (Additional file 1: Figure S8).

The higher consistency of seed mediated off-target effects suggests that although the specific effects of each individual shRNA within a shRNA family might differ in terms of the target profile of down-regulated genes, averaging over the shRNA members is likely to capture the combined essentiality of the shared off-target profile of genes, determined by its identical seed sequence. The phenotypic effect of down-regulating multiple off-targets compared to a single intended on-target gene is likely to be similar due to the perturbation effect on many players in a cellular system. In contrast, summary estimates from conventional on-target gene essentiality profiles are likely to have more variation due to the variable effects of each shRNA against its intended target. Based on our observations, we therefore recommend the use of multiple shRNAs with identical seed sequences when designing future genome-wide shRNA libraries, as this enables one to accurately estimate the seed-level essentiality scores. Sampling over multiple shRNAs with the same seed sequence to estimate the seed essentiality, followed by modeling the target genes based on seed sequence complementarity, should allow us to derive more accurate geneESs in such improved screens.

The role of seed-mediated effects has been studied previously in various biological contexts other than cancer, including host factors required for pathogen infections [30], regulators of TRAIL-induced apoptosis [38], and genes responsible for spindle assembly checkpoint [33]. Various computational methods for modeling seed-mediated effects in siRNA screens have also been designed to identify off-target genes/pathways [33–35, 37]. However, these existing methods do not account for other factors that are specific to shRNA screens, such as heterogeneous processing of shRNAs. It has been observed previously that shRNAs expressed under different promoter architecture, pol II or pol III, yield mature guide strands that are shifted in their sequence, resulting in altered seed sequences [55]. Instead, we focused on enrichment of the on-target activity of shRNAs in the cancer context and derived better estimates of gene-level essentiality phenotypes that can be adopted and implemented easily for wider use.

As a straightforward outcome of these results, we provide a practical solution for cleaning up the existing genome-wide shRNA datasets by effectively removing those shRNAs with seed sequences having a higher likelihood of off-target effects from the downstream post-screening data analysis. In these analyses, we made use of previously identified determinants of targeting proficiency of miRNAs and siRNAs [41, 49, 50], namely SPS and TA. As a novel contribution, we quantitatively showed their relevance to increased consistency of genome-wide shRNA screening data. We promote the use of these practical guidelines (summarized in Additional file 1: Figure S9) with the aim of addressing the current problems of off-target effects and to make the most of the existing and emerging genome-wide shRNA screens. These guidelines should be updated in the future once more actionable insights into the shRNA biology become available; for instance, information on the frequency of seed complementary sites in the full transcript, not only restricted to 3′ UTR, as well as taking into account pairing based on the 3′ region of the shRNA sequence, might further improve the prediction of relevant off-target sites.

To demonstrate the potential of this strategy in the identification of novel genetic interaction partners of major cancer driver genes, we experimentally validated the predicted synthetic lethality partners of PIK3CA using CRISPR/Cas9 knockout screening as a case study of potential anticancer treatments for PIK3CA driven cancers. One of the confirmed partners, PKN3, has been reported to be involved in tumor angiogenesis and metastasis [56], and having a role as a downstream effector of PI3K signaling [57]. Similarly, the other confirmed partner, HMX3, is an activated transcription factor regulator in the HER2 subtype of breast cancer [58]. Although these examples demonstrated the potential of this strategy to (i) increase the overall reproducibility of pan-cancer GI detections and (ii) find novel SL partners of major cancer drivers in a particular cell context (here, MCF10A), the practical implications of these findings for identification of druggable synthetic lethal partners for targeted therapeutic interventions need to be validated in further pre-clinical or clinical studies.

These results on the reproducibility of genome-wide shRNA screens resemble the recent debate about the consistency of large-scale drug response profiling in cancer cell lines, where the first comparative study reported poor consistency in the drug response phenotypes between two laboratories [59]. However, follow-up analyses demonstrated that when robust response calculations are used, and when the evaluation metrics are aligned with the objectives of the functional profiling, acceptable consistency can be achieved, provided that the screening assays and experimental protocols are similar enough [60–62]. Off-target effects have also been observed with the CRISPR/Cas9 system [63], making these lessons likely useful also for improving future CRISPR/Cas9 study designs. A number of computational tools have already been implemented for off-target prediction and gene essentiality scoring in genome-wide CRISPR/Cas9 knockout screens, which make use of similar concepts as those for RNAi experiments [63–65]. Distinct advantages and limitations of both RNAi and CRISPR/Cas9 screening technologies seem to remain, making their complementary use warranted in future loss-of-function profiling studies [66].

## Conclusions

Despite the pervasive off-target effects in genome-wide shRNA screens, we observed a moderate between-study consistency that can be improved by controlling for factors that determine off-target propensity. After controlling for such factors in the post-processing of genome-wide shRNA screens, one can improve the reproducibility of identified genetic interactions and synthetic lethal partners of cancer driver genes, a finding that has direct implication for better development of targeted anticancer treatment options and studying the functional landscape of cancer cells.

## Additional files

Additional file 1: Figure S1. Correlation based on shESs in high data quality cell lines. **Figure S2.** Examples of seed essentiality (seedES) calculations in an artificial dataset. **Figure S3.** Rank correlation (ρ) for high data quality cell lines based on shES and seedES over all shRNA family sizes. **Figure S4.** Reproducibility of the seed essentiality scores with increasing shRNA family size of seed sequences. As shown in Fig. 3, we added the *gray trace* indicating the correlation based on the average of correlations from all positions. **Figure S5.** Heatmap of average Spearman correlation of seedES scores with increasing family size, between the matched cell lines, by considering different positions along the shRNA molecule as the seed sequence. **Figure S6.** As shown in Fig. 5, the number of overlapping SL partners of major cancer driver genes observed in both datasets, before and after cleaning, where the cleaning was based on removal of shRNAs with a high tendency for off-target seed effects (defined by SPS and TA properties of seed sequences; Fig. 4). **Figure S7.** GARP-based geneES for PKN3 and HMX3 before and after cleaning in PIK3CA mutant and wild-type (*WT*) cell lines, separately for the Achilles 2.4 and COLT-cancer datasets. **Figure S8.** Density plots of geneES scores for all the genes and gold-standard constitutive core essential (*CCE*) genes. Gene-specific phenotypes were calculated based on gespeR and GARP scores in both Achilles and COLT-Cancer datasets, respectively. **Figure S9.** A stepwise procedure for cleaning genome-wide shRNA datasets. **Figure S10.** Baseline reproducibility between the Achilles 2.4 and BFG genome-wide shRNA screens. **Figure S11.** Reproducibility of Achilles 2.4 and BFG genome-wide screens at the level of shRNAs, on-target genes, and off-target seeds. **Figure S12.** Reproducibility of seed essentiality scores with increasing shRNA family size of seed sequences in additional datasets. **Figure S13.** Reproducibility of Achilles 2.4 and BFG datasets after accounting for seed sequence properties. (DOCX 1588 kb)

Additional file 2: Table S1. Sequences of sgRNAs used against HMX3 and PKN3. (DOCX 13 kb)

## Abbreviations

GARP: Gene activity rank profile
geneES: Gene essentiality score
GI: Genetic interaction
heptamer12–18ES: Heptamer 12–18 essentiality score
miRNA: micro RNA
NGS: Next-generation sequencing
QC: Quality control
RIGER: RNAi gene enrichment ranking
RNAi: RNA interference
seedES: Seed essentiality score
sgRNA: single-guide RNA
shES: shRNA essentiality score
shRNA: Short hairpin RNA
siRNA: Small interfering RNA
SL: Synthetic lethal
SPS: Seed pairing stability
TA: Target site abundance
UTR: Untranslated region
