# Supplementary material for: Seed-effect modeling improves the consistency of genome-wide loss-of-function screens and identifies synthetic lethal vulnerabilities in cancer cells
Source: Genome Med. 2017 Jun 1;9:51. doi: 10.1186/s13073-017-0440-2 (PMC5452371; doi:10.1186/s13073-017-0440-2)
Supplement: Supplementary file 1 — Correlation based on shESs in high data quality cell lines. Figure S2. Examples of seed essentiality (seedES) calculations in an artificial dataset. Figure S3. Rank correlation (ρ) for high data quality cell lines based on shES and seedES over all shRNA family sizes. Figure S4. Reproducibility of the seed essentiality scores with increasing shRNA family size of seed sequences. As shown in Fig. 3, we added the gray trace indicating the correlation based on the average of correlations from all positions. Figure S5. Heatmap of average Spearman correlation of seedES scores with increasing family size, between the matched cell lines, by considering different positions along the shRNA molecule as the seed sequence. Figure S6. As shown in Fig. 5, the number of overlapping SL partners of major cancer driver genes observed in both datasets, before and after cleaning, where the cleaning was based on removal of shRNAs with a high tendency for off-target seed effects (defined by SPS and TA properties of seed sequences; Fig. 4). Figure S7. GARP-based geneES for PKN3 and HMX3 before and after cleaning in PIK3CA mutant and wild-type (WT) cell lines, separately for the Achilles 2.4 and COLT-cancer datasets. Figure S8. Density plots of geneES scores for all the genes and gold-standard constitutive core essential (CCE) genes. Gene-specific phenotypes were calculated based on gespeR and GARP scores in both Achilles and COLT-Cancer datasets, respectively. Figure S9. A stepwise procedure for cleaning genome-wide shRNA datasets. Figure S10. Baseline reproducibility between the Achilles 2.4 and BFG genome-wide shRNA screens. Figure S11. Reproducibility of Achilles 2.4 and BFG genome-wide screens at the level of shRNAs, on-target genes, and off-target seeds. Figure S12. Reproducibility of seed essentiality scores with increasing shRNA family size of seed sequences in additional datasets. Figure S13. Reproducibility of Achilles 2.4 and BFG datasets after accounting for seed sequence propert [file 13073_2017_440_MOESM1_ESM.docx]

**Figure S1:** Boxplot of correlation based on shESs in high data quality cell lines (x-axis). **(A)** Achilles 2.4 cell lines compared with the complementary set of non-matching cell lines in COLT-cancer. Red dots indicate the correlation with the matching cell line in COLT-cancer. **(B)** COLT-cancer cell lines compared with the complementary set of non-matching cell lines in Achilles 2.4. Red dots indicate the correlation with the matching cell line in Achilles 2.4.

**Figure S2:** Schematic examples of seed essentiality (seedES) calculations in an artificial dataset of 17 shRNAs. For each matching cell line, in the common dataset of 46,474 shRNAs identified by their clone identifier, the shRNAs are grouped by their seed sequence identity in guide strand. SeedESs are calculated by averaging over shES of all the shRNAs having an identical seed sequence, represented by the same color as the seed sequence in the table. shRNA family size of a given seed is the number of shRNAs having the same sequence. In heptamer12-18ES calculations, instead of the sequence identity from 2-8 positions, the shRNAs were grouped based on the sequence identity from 12-18 positions and then averaged. For the permutation analysis, we shuffled the shES and seed sequence mapping in the total dataset and calculated the permuted seedES as an average over 1000 random permutations.

**Figure S3:** Scatterplots showing rank correlation (ρ) for high data quality cell lines based on **(A)** shES vs. seedES over all shRNA family sizes, **(B)** shES vs. heptamer12-18ES for all family sizes >= 5.

**Figure S4:** As shown in Figure 3, we added the gray trace that indicates the correlation based on the average of correlation from all positions. Similar to the analysis for Heptamer 12-18ES, we repeated the same analysis for all positions of shRNAs and calculated hepatmerES scores at each interval and estimated the correlation between the screens based on these scores. Finally, the correlation estimates at all other intervals except for the seed interval, 2-8, were averaged for each cell line and plotted as the gray trace.

**Figure S5:** Heatmap of average Spearman correlation of seedES scores with increasing family size, between the matched cell lines, by considering different positions along the shRNA molecule as the seed sequence. Seed positions at 5’ UTR end of the shRNA molecule tend to show increasing correlation, especially with larger family size, suggesting the heterogeneous processing of shRNAs contributes substantially to the observed variability of the shRNA screens.

**Figure S6:** As shown in Figure 5, the number of overlapping SL partners of major cancer driver genes observed in both datasets, before and after cleaning, where the cleaning was based on removal of shRNAs with high tendency of off-target seed effects (defined by SPS and TA properties of seed sequences, see Figure 4). SL partners were defined based on one-sided Wilcoxon rank sum test (p <0.03). The statistical significance of the difference in the number of overlapping SL partners, before and after cleaning, was tested with one-sided Wilcoxon signed rank test. The color-coding of the drivers indicate the loss-of-function (black), activating (red) or unclassified (grey) status of the driver mutations, as extracted from IntoGen (https://www.intogen.org/)

**Figure S7:** Boxplots of GARP based geneES for PKN3 (A, B) and HMX3 (C, D) before and after cleaning in PIK3CA mutant and wild-type (WT) cell lines, separately for the Achilles 2.4 (A, C) and COLT-cancer datasets (B, D). Statistical significance in geneES between mutant and WT cells was tested with Wilcoxon rank sum test (ns, p>0.05). In general, we observed that cleaning boosts the differential gene essentiality between PIK3CA mutant and WT cells (i.e., synthetic lethality) for both PKN3 and HMX3 partners, especially in the COLT-cancer dataset.

**Figure S8:** Density plots of geneES scores for all the genes and gold-standard constitutive core essential (CCE) genes. Gene-specific phenotypes (A, B) were calculated based on gespeR and GARP scores (C, D), in both Achilles and COLT-Cancer datasets, respectively.

**Figure S9:** A stepwise procedure for cleaning genome-wide shRNA datasets.

**Description of Breast Functional Genomics dataset:**

The Breast Functional Genomics (BFG) dataset was generated from a genome-wide shRNA screen of 77 breast cancer cell lines, out of which 12 cell lines were common with Achilles 2.4 dataset. Similar to the Achilles 2.4, the BFG screen studied ~78k shRNAs from The RNAi Consortium library, and shRNA abundance was measured by NGS at least in 3 time points during growth phase. The raw data were deconvoluted and processed further to estimate the effect of each individual shRNA on cell proliferation. Briefly, scoring of shRNAs in the BFG study was done by averaging of the fold changes in read counts of shRNAs between the three time points, followed by calculating the GARP scores, similarly as was done for COLT-cancer study. The same research group, who performed COLT-Cancer study, also performed the BFG study; however, breast cancer cell lines were the primary focus of the BFG study.

**Figure S10:** Baseline reproducibility between the Achilles 2.4 and BFG genome-wide shRNA screens. **(A)** Overlap in shRNAs, target genes and cell lines screened in the Achilles 2.4 and BFG studies. Based on sequence identity, we found 47,175 shRNAs were commonly profiled in the two studies. **(B)** Inter-study correlation (ρ) for shES across matched cell lines between Achilles 2.4 and BFG datasets. The black dashed line indicates average correlation (ρ = 0.53) over the 12 cell lines shared between the BFG and Achilles 2.4.

**Figure S11:** Reproducibility of Achilles 2.4 and BFG genome-wide screens at the level of shRNAs, on-target genes, and off-target seeds. Comparison of rank correlation (ρ) between Achilles 2.4 and BFG over the 12 cell lines, where each panel compares the between-study correlation of shRNA essentiality scores (shESs, x-axis) against the correlation calculated based on **(A)** GARP based geneES, and **(B)** seed essentiality scores (SeedES), calculated for seeds with shRNA family size larger than 5. As observed in the original analyses (Fig. 2), the on-target GARP based gene essentiality score did not improve the consistency beyond the shES-level comparison, whereas accounting for off-target effects based on SeedES improved the consistency among the matching cell lines. Statistical significance of correlation differences was assessed with paired *t*-test.

**Figure S12:** Reproducibility of the seed essentiality scores with increasing shRNA family size of seed sequences. Average rank correlation (ρ), with standard error of mean over the 12 cell lines (error bars), calculated based on SeedES as a function of shRNA family size (x-axis). shRNAs sharing the same seed sequence belong to the same shRNA family. Red trace indicates the observed correlation based on seed region. Blue trace indicates the correlation based on heptamer12-18 ES for 12-18 positions. Black trace indicates correlations based on 1000 permutations over the seed – shRNA mapping. SeedES-based inter-study correlation reached its maximum at family size of 13 (ρ=0.71), suggesting that the consistency between the studies increases when off-target effects are more accurately estimated using larger family size. Asterisk indicates statistically significant differences in correlations (*p* < 0.05, paired t-test), and their colors indicate the distribution against which the comparison was done.

**Figure S13:** Reproducibility of Achilles 2.4 and BFG datasets after accounting for seed sequence properties. Rank correlation (ρ) over the 12 cell lines for shES of shRNAs with strong or weak SPS, or, low or high TA. Asterisk denotes statistically significant differences in correlation (*p* < 0.05, paired *t*-test). Strong SPS was defined as top 10% percentile (SPS < -9.82), and weak SPS as bottom 10% percentile (SPS > -5.16). Low TA > 3.72 and high TA < 2.89 were defined similarly, as shown on top of each panel.
