# Supplementary material for: Seed-effect modeling improves the consistency of genome-wide loss-of-function screens and identifies synthetic lethal vulnerabilities in cancer cells
Source: Genome Med. 2017 Jun 1;9:51. doi: 10.1186/s13073-017-0440-2 (PMC5452371; doi:10.1186/s13073-017-0440-2)
Supplement: Supplementary file 2 — Sequences of sgRNAs used against HMX3 and PKN3. (DOCX 13 kb) [file 13073_2017_440_MOESM2_ESM.docx]

**Additional file 2: Table S1**

HMX3-g1

GGCAGTGGCTCCGGAGAGTCG

HMX3-g2

GCGACTCCGAGGAAAGCAAAA

HMX3-g3

GTGAAAGTCCAGAGAAGAAGC

PKN3-g1

GGAAAGAGCTGAAGATCAAGG

PKN3-g2

GACTGCACGTTGAGGCAGCTG

PKN3-g3

GGGTGCTGGCTGTGCTAAAGG

NoTargetControl-GFP-g1

GCGTCGCCGTCCAGCTCGACC
